# Supplementary material for: Trends in Mortality Due to Malignant Neoplasms of Female Genital Organs in Poland in the Period 2000–2021—A Population-Based Study
Source: Cancers (Basel). 2024 Mar 3;16(5):1038. doi: 10.3390/cancers16051038 (PMC11154286; doi:10.3390/cancers16051038)
Supplement: Supplementary file 1 [file cancers-16-01038-s001.zip › Suplement 1.pdf]

Table S1. Deaths due to the malignant neoplasms of female genital organs in the years 2000-2021

**Causes of death**	**2000**	**2001**	**2002**	**2003**	**2004**	**2005**	**2006**	**2007**	**2008**	**2009**	**2010**	**2011**	**2012**	**2013**	**2014**	**2015**	**2016**	**2017**	**2018**	**2019**	**2020**	**2021**
**Number**																						
Malignant neoplasms of female genital organs (C51-C58) including:	5841	5743	5704	5865	5863	5870	5926	6277	6148	6147	6193	6156	6086	6401	6462	6828	6553	6763	6941	7006	6811	6412
Vulva cancer (C51)	195	207	203	213	194	169	186	280	265	232	273	270	273	303	267	315	326	315	319	371	367	342
Vagina cancer (C52)	66	73	48	61	80	92	67	81	55	53	45	44	57	63	42	65	64	56	68	60	42	59
Cervix uteri cancer (C53)	1987	1826	1855	1825	1819	1796	1824	1907	1745	1748	1735	1656	1669	1669	1628	1585	1570	1609	1593	1569	1511	1361
Corpus uteri cancer (C54)	808	776	757	783	794	770	814	848	952	969	1042	1085	1162	1243	1280	1690	1600	1761	1781	1859	1811	1646
Uterus unspecified cancer (C55)	460	437	430	469	427	439	401	376	345	378	288	232	213	246	255	168	169	152	167	149	174	131
Ovary cancer (C56)	2033	2152	2171	2271	2273	2357	2390	2485	2507	2510	2547	2558	2432	2603	2678	2768	2639	2670	2829	2777	2688	2637
Other female genital organs cancer (C57)	292	271	238	243	276	247	244	298	279	256	261	311	278	273	309	236	185	200	182	221	217	235
Placenta cancer (C58)	0	1	2	0	0	0	0	2	0	1	2	0	2	1	3	1	0	0	2	0	1	1
**SDR (per 100,000)**																						
Malignant neoplasms of female genital organs (C51-C58) including:	36.7	35.3	34.8	35.3	34.6	34.1	34.0	35.4	34.4	33.9	33.4	32.9	31.9	33.0	33.0	34.3	32.7	33.0	33.5	33.3	32.1	30.1
Vulva cancer (C51)	1.4	1.4	1.3	1.4	1.2	1.1	1.1	1.7	1.5	1.3	1.5	1.5	1.5	1.6	1.4	1.6	1.6	1.5	1.5	1.7	1.7	1.6
Vagina cancer (C52)	0.4	0.5	0.3	0.4	0.5	0.6	0.4	0.5	0.3	0.3	0.3	0.2	0.3	0.3	0.2	0.3	0.3	0.3	0.3	0.3	0.2	0.3
Cervix uteri cancer (C53)	12.0	10.7	10.9	10.6	10.3	10.0	10.2	10.5	9.5	9.4	9.1	8.7	8.6	8.5	8.2	7.9	7.8	7.8	7.6	7.5	7.2	6.4
Corpus uteri cancer (C54)	5.3	4.9	4.9	4.9	4.9	4.6	4.9	5.0	5.5	5.5	5.8	5.9	6.3	6.5	6.6	8.5	8.0	8.5	8.6	8.8	8.5	7.6
Uterus unspecified cancer (C55)	3.0	2.8	2.8	3.0	2.6	2.7	2.4	2.2	2.0	2.1	1.6	1.3	1.1	1.3	1.3	0.9	0.8	0.7	0.8	0.7	0.9	0.6
Ovary cancer (C56)	12.7	13.2	13.1	13.5	13.3	13.7	13.6	13.9	14.0	13.8	13.7	13.6	12.7	13.4	13.7	14.0	13.2	13.1	13.8	13.3	12.8	12.5
Other female genital organs cancer (C57)	2.0	1.8	1.6	1.6	1.7	1.5	1.5	1.7	1.5	1.4	1.4	1.7	1.5	1.4	1.6	1.2	0.9	1.0	0.9	1.0	1.0	1.1
Placenta cancer (C58)	0.0	0.0	0.0	0.0	0.0	0.0	0.0	0.0	0.0	0.0	0.0	0.0	0.0	0.0	0.0	0.0	0.0	0.0	0.0	0.0	0.0	0.0
