# Supplementary material for: Trends in Mortality Due to Malignant Neoplasms of Female Genital Organs in Poland in the Period 2000–2021—A Population-Based Study
Source: Cancers (Basel). 2024 Mar 3;16(5):1038. doi: 10.3390/cancers16051038 (PMC11154286; doi:10.3390/cancers16051038)
Supplement: Supplementary file 1 [file cancers-16-01038-s001.zip › Suplement 2.pdf]

Supplement 2.

Table S2. Morbidity on the three most frequent malignant neoplasms of female genital organs in the years 2000-2020

	2000	2001	2002	2003	2004	2005	2006	2007	2008	2009	2010	2011	2012	2013	2014	2015	2016	2017	2018	2019	2020
**Number**																					
Cervix uteri cancer (C53)	3733	3671	3711	3465	3459	3381	3345	3577	3433	3198	3140	3028	2840	2917	2859	2776	2689	2562	2425	2482	1950
Corpus uteri cancer (C54)	3696	3888	4027	4155	4410	4454	4594	4890	5013	5326	5231	5388	5520	5796	6041	6360	6405	6087	6184	6188	5290
Ovary cancer (C56)	3201	3362	3391	3462	3394	3553	3519	3473	3553	3607	3628	3551	3572	3646	3733	3777	3692	3743	3705	3685	2960
**SMR (per 100,000)**																					
Cervix uteri cancer (C53)	21.3	20.6	20.7	19.3	18.9	18.3	18.2	19.1	18.1	16.9	16.3	15.7	14.5	14.8	14.4	13.8	13.3	12.6	11.8	11.9	9.4
Corpus uteri cancer (C54)	22.9	23.9	24.3	24.8	25.8	25.7	26.2	27.4	27.8	29.0	28.0	28.6	28.9	30.0	30.7	32.0	32.0	30.1	30.2	29.9	25.3
Ovary cancer (C56)	19.0	19.7	19.6	19.9	19.3	20.0	19.5	19.1	19.3	19.4	19.3	18.7	18.6	18.7	19.0	19.1	18.5	18.6	18.3	18.0	14.9

SMR – Standardized Morbitity Rates

Source: Polish National Cancer Registry https://onkologia.org.pl/en/report

Table S3. Time trends of standardized morbidity rates (SMR) on the three most frequent malignant neoplasms of female genital organs in the years 2000-2020– joinpoint regression analysis

	Number of joinpoints	Years	APC (95% CI)	AAPC (95% CI)
Cervix uteri cancer (C53)	1	2000-2018<br>2018-2020	-3.0* (-3.3; -2.7)<br>-12.0* (-20.1; -3.0)	-3.9* (-4.8; -3.0)
Corpus uteri cancer (C54)	1	2000-2016<br>2016-2020	2.0* (1.7; 2.4)<br>-4.5* (-7.0; -1.9)	0.7* (0.1; 1.2)
Ovary cancer (C56)	1	2000-2018<br>2018-2020	-0.3* (-0.5; -0.1)<br>-9.2* (-13.7; -4.6)	-1.2* (-1.7; -0.8)
